# Complementary Effects of Dark Septate Endophytes and *Trichoderma* Strains on Growth and Active Ingredient Accumulation of *Astragalus mongholicus* under Drought Stress

**DOI:** 10.3390/jof8090920

**Published:** 2022-08-30

**Authors:** Min Li, Yanfang Ren, Chao He, Jiaojie Yao, Miao Wei, Xueli He

**Affiliations:** 1 School of Life Sciences, Hebei University, Baoding 071002, China; 2 Key Laboratory of Microbial Diversity Research and Application of Hebei Province, No. 180, Wusidong Rd., Baoding 071002, China; 3 Institute of Medicinal Plant Development, Chinese Academy of Medical Sciences & Peking Union Medical College, Beijing 100193, China

**Keywords:** dark septate endophyte, *Trichoderma*, co-inoculation, drought stress, *Astragalus mongholicus*

## Abstract

Drought is a major abiotic stress factor affecting plant growth and production, while utilizing beneficial endophytic fungi is one of the most promising strategies for enhancing plant growth and drought tolerance. In the current study, a pot experiment was conducted to investigate the beneficial effects of dark septate endophyte (DSE) (*Macrophomina pseudophaseolina*, *Paraphoma radicina*) and *Trichoderma* (*Trichoderma* *afroharzianum*, *Trichoderma* *longibrachiatum*) inoculum on *Astragalus mongholicus* grown in sterile soil under drought stress, alone, or in combination. The addition of *Trichoderma* enhanced the DSE colonization in roots regardless of the water condition. Under well-watered conditions, *M. pseudophaseolina* inoculation significantly enhanced the biomass and root length of *A. mongholicus*. The two DSE and *Trichoderma* inoculum significantly improved calycosin-7-O-β-D-glucoside content. However, *M*. *pseudophaseolina* + *T.* *afroharzianum* inoculation better promoted root growth, whereas co-inoculation had higher active ingredient contents compared with single inoculation, except for *P. radicina* + *T.* *afroharzianum*. Under drought stress, DSE and *Trichoderma* inoculum significantly improved root biomass, root length, calycosin-7-O-β-D-glucoside content, and activities of nitrate reductase and soil urease. *P. radicina* + *T. afroharzianum* and *P. radicina* + *T.* *longibrachiatum* better increased root length, and all combinations of DSE and *Trichoderma* had a greater impact on the increase in formononetin content compared with the single treatments. Additionally, *Trichoderma* relies on antioxidant enzymes, growth hormones, and the redox system (ascorbic acid–glutathione) to resist drought, while DSE strains have an additional osmotic regulation system in addition to the drought resistance function possessed by *Trichoderma*, and the effect of co-inoculation (especially *M*. *pseudophaseolina* + *T.* *longibrachiatum* and *P. radicina* + *T. afroharzianum*) on plant physiological parameters was greater than that of single inoculation. This study provides a new research direction for the effects of DSE and *Trichoderma* on medicinal plant cultivated in dryland.

## 1. Introduction

Although drought can destroy the soil structure, reduce soil productivity, limit the absorption of water and nutrients, cause an imbalance in plant infiltration and redox, and eventually lead to plant death [1,2], plant–endophyte symbionts have developed a series of regulatory mechanisms in response to environmental stress during the course of evolution [3]. Endophytic fungi can augment the secretion of host plant endogenous hormones such as ascorbic acid (ASA), proline, and enzymes such as superoxide dismutase, peroxidase, etc., which constitute the total antioxidant system of cells to adapt to drought conditions [4,5]. Therefore, using beneficial endophytic fungi to maintain plant health and quality is an effective way to improve crop yield and productivity. This requires us to select fungal species that can adapt to drought conditions and host plants that can efficiently symbiose with them.

Dark septate endophytes (DSE), a major group of endophytes of Ascomycetes, are characterized by melanized septate hyphae and microsclerotia structures in the roots [6]. These fungi inhabit the root cortex and vascular tissues of healthy plants, especially those under stressful habitats such as drought, salt, and heavy metal stress [7]. In the course of evolution, the adaptive strategies of DSE strains to stress environments ranged from morphological to physiological and biochemical characteristics. Melanin produced by DSE during the growth process can combine with oxygen free radicals to enhance DSE resistance to environmental stresses such as drought [8]. Studies have shown that DSE can regulate plant endogenous hormone levels [9], and the mycelium extended by DSE can adjust the plant root morphological structure and expand the root network to enhance the water absorption and nutrient transport of host plants under drought stress [10,11]. *Trichoderma* is a nonpathogenic rhizosphere-colonizing fungus, whose hyphae coil around the roots to penetrate the root epidermis and settle between the epidermis and cortex cells [12]. *Trichoderma* can effectively protect plants from abiotic stresses by stimulating root growth, improving nutrient absorption, regulating endogenous plant hormones, and enhancing antioxidants to remove reactive oxygen species, thus improving the stress tolerance of plants. *T. asperelloides* significantly contributed to shoot weight of tomato under water deficit conditions [13]. *T. simmonsii*, *T. virens*, and *T. spirale* highlighted the protection of wheat under drought conditions by activating the mechanism of antioxidant enzymes and increasing the content of hormones in wheat [14,15]. In addition, *Trichoderma* can enlarge the contact area between the rhizosphere and the soil, increase the secretion of extracellular enzymes such as phosphatase, urease, sucrase, and organic acids to activate the nutrients in the soil, promote the absorption of nutrients, and improve the circulation and utilization of nutrients in the soil [16,17]. However, existing studies on beneficial fungi promoting plant growth mainly focus on single inoculation, and mixed inoculation leads to stronger resistance to stress than single inoculation [18,19]. The co-inoculation of *Trichoderma* spp., *Glomus* spp., and *Bacillus* spp. not only more effectively protected the plants from pathogenic microbes, but stimulated the plant’s defense mechanisms to protect it from the adverse environment [20,21]. The combined application of *Trichoderma* and ectomycorrhizal fungi effectively increased the antioxidant capacity of *Pinus sylvestris* seedlings [22].

*Astragalusmongholicus* Bunge is a perennial legume herb whose roots are important medicinal materials for clinical treatment in China because of their pharmacological actions such as enhancing immunity, lowering blood pressure, and anti-inflammation [23]. Calycosin-7-o-β-d-glucoside and formononetin, as important secondary metabolites of flavonoids in *Astragalus* roots, are among the indicator components for evaluating the quality of *Astragalus* roots [24]. At present, it is urgent to improve the quality and drought resistance of *A. mongholicus* in farmland cultivation and promote the sustainable development of medicinal plants. Existing studies have found that *Rhizobium* sp., *Sinorhizobium* sp., and their combination inoculation significantly increased the weight of *A. membranaceus* seedlings and the accumulation of flavonoids [25]. *Pseudomonas poae* inoculation had beneficial effects on root biomass and calycosin-7-O-glucoside accumulations of *A. mongholicus* by pouring 20% PEG6000 to mimic drought stress [26]. Hence, understanding the interactions between *A. mongholicus* and beneficial microbes is essential to benefit from symbiotic mechanisms.

Although it was found that *Trichoderma* was dominant in the rhizosphere of medicinal plants, and DSE could also colonize the roots of medicinal plants [9,27], there are not comprehensive studies of the effects of the combined inoculation of DSE and *Trichoderma* on the nodulation, plant growth, accumulation of secondary metabolites, and soil nutrients of *A. mongholicus* under drought stress. The objective of this study was, therefore, to explore the potential effects of DSE inoculation (*Macrophomina pseudophaseolina*, *Paraphoma radicina*), *Trichoderma* inoculation (*Trichoderma afroharzianum*, *Trichoderma longibrachiatum*), as well as dual inoculation of DSE and *Trichoderma* strains, on the growth of *A. mongholicus* grown on arid land. Here, we investigated the effects of single and dual inoculation under drought conditions on (I) root structure, (II) plant biomass, (III) active ingredients, (IV) physiological parameters, and (V) soil physicochemical properties. These data can demonstrate whether DSE and *Trichoderma* can withstand drought stress and whether they have the potential to improve the stress resistance and symbiosis of *A. mongholicus* in drought-affected lands.

## 2. Materials and Methods

### 2.1. Fungal Isolates and Plant Materials

The two DSE strains used in the experiment were obtained from *A. mongholicus* roots and two *Trichoderma* strains were isolated from the rhizosphere soil of *Bacopa monnieri* and *Scutellaria baicalensis* grown naturally in farmland habitat in Anguo city, Hebei Province, China, and were preserved in the culture collection of the Laboratory of Mycorrhizal Biology, Hebei University, China. These fungal species were identified based on morphological characteristics (Figure S1) and phylogenetic analysis of internal transcribed spacer (ITS) and translation elongation factor 1-α (tef1) gene sequences (Figure S2). All isolates were cultured on the PDA medium for two weeks under dark conditions at 27 °C for subsequent experiments.

Sterile water (5 mL) was placed in a Petri dish containing mature *Trichoderma*, and the conidia produced were gently scraped and collected with a sterile spatula. The spore suspensions obtained were used as inoculum after measuring the spore concentration (ca. 1 × 10^7^ CFU mL^−1^) in a hemocytometer.

Mature seeds of *A. mongholicus* were collected from the Anguo Medicine Planting Site in Hebei Province, China, and stored at 4 °C.

### 2.2. Inoculation Assay

The inoculation experiment was conducted using a completely randomized factorial design (3 DSE inoculations × 3 *Trichoderma* inoculations × 2 water conditions) with five replicates. The three DSE inoculations were inoculation *M*, inoculation *P*, and non-inoculated control. The three *Trichoderma* inoculations were inoculation *A*, inoculation *L*, and non-inoculated control. Dual inoculations were inoculation *MA*, inoculation *ML*, inoculation *PA*, and inoculation *PL*. The two water conditions were well-watered (WW, 65% field water capacity) and drought stress (DS, 30% field water capacity). There were two plants per pot, thus accounting for a total of 90 experimental pots.

Seeds of *A. mongholicus* were surface-sterilized with 50% (*v/v*) sulfuric acid for 14 min and then 5% (*v/v*) sodium hypochlorite for 10 min. The sterilized seeds were rinsed six times with sterile water and then germinated in a constant temperature and humidity incubator at 27 °C for one week. The seedlings were transplanted to sterile plastic pots (9.5 cm diameter, 11.5 cm height) containing 500 g sterile growth medium, which was a 1:1 (*w/w*) mixture of sand and soil (<2 mm) collected from the growing site of *A. mongholicus* and autoclaved for 120 min at 121 °C. The growth medium contained 21.38 mg g^−1^ organic matter, 51.90 μg g^−1^ available nitrogen, and 18.62 μg g^−1^ available phosphorus. For DSE inoculation, two 5 mm discs were cut from the DSE colony and inoculated at 1 cm to the roots of *A. mongholicus* seedlings, and the sterile PDA medium without fungus was inoculated as the control treatment. After 30 days, the seedlings were irrigated with 30 mL of *Trichoderma* strain spore suspension and an equal amount of sterilized liquid was poured onto the control seedlings [28]. All the above inoculation procedures were carried out in a completely sterile environment, and all the pots were kept in a growth chamber (10 h photoperiod, 27 °C/22 °C (day/night), 60% mean relative humidity). Two months after sowing, half of the seedlings (both control and inoculation treatments) were subjected to WW treatment, and the other half were subjected to DS treatment. The *A. mongholicus* seedlings were harvested 120 days after sowing. The fresh weights of shoots and roots, root colonization, morphological traits, and active ingredient contents, as well as physiological and soil parameters, were measured 120 days after sowing.

### 2.3. Plant Growth Parameters

Plant shoots and roots were harvested, and the roots were gently washed with deionized water to remove the surface soil. Individual root sections were completely floated in plexiglass trays containing deionized water (approximately 0.3 cm deep) and scanned by a desktop scanner (EPSON Perfection V800 Photo; Epson, Nagano, Japan). The total root length and average root diameter were determined by the WinRHIZO image analysis system.

### 2.4. Physiological Parameter of Plant Leaves

The soluble protein concentration was determined by the Coomassie Brilliant Blue G-250 reagent [29]. Fresh leaves (0.5 g) were homogenized in ice-cold phosphate buffer (50 mM/5 mL, pH 7.0) in an ice bath. The homogenate was centrifuged at 4000× *g* for 10 min at 4 °C to collect the supernatant. The absorbance of the supernatant was measured at 595 nm using a spectrophotometer (752 N model, Shanghai INESA Instrument Analytical Instruments Co., Ltd., Shanghai, China). Bovine serum albumin was used as a standard.

The proline content was determined based on the method of Bates [30]. Sulfosalicylic acid solution (3%, 5 mL) was used to homogenize fresh leaves (0.5 g) to a homogenate that was filtered through filter paper after being heated in boiling water for 10 min. The filtrate (2 mL) was reacted with acid ninhydrin (2 mL) and glacial acetic acid (2 mL) for 30 min at 100 °C, then terminated in an ice bath. Toluene (4 mL) was used to extract the reaction mixture, and the absorbance was read at a wavelength of at 520 nm.

The glutathione (GSH) content was determined by the dithio-dinitrobenzoic acid (DTNB) method [31]. Fresh leaves (0.5 g) were ground into a homogenate in 5% of 5 mL trichloroacetic acid and centrifuged at 10,000× *g* for 10 min. Then, 0.25 mL supernatant was added to 2.6 mL NaH_2_PO_4_ (150 mm/L) and 0.15 mL DTNB, and the reaction was kept at 30 °C for 5 min. The absorbance was measured at 412 nm with a spectrophotometer.

SOD activity was measured by the photochemical reduction method [32]. A potassium phosphate buffer (50 mM/5 mL, pH 7.8) containing chilled EDTA (0.2 mM) and polyvinylpyrrolidone (2%, *w/v*) was used to homogenize fresh leaves (0.5 g) in an ice bath. The supernatant was collected after the homogenate was centrifuged at 15,000 rpm for 30 min before enzymatic assays. The photochemical reduction of nitroblue tetrazolium was determined at a wavelength of 560 nm using a spectrophotometer.

### 2.5. Physiological Parameter and Active Ingredients of Plant Roots

Nitrate reductase (NR) activity was assessed by the in vitro method to determine the nitrite nitrogen content [33]. GSH content was determined by the DTNB method (see above). The content of ASA in roots was determined by referring to Kampfenkel [34]. The root indole-3-acetic acid (IAA) concentration was determined by an ELISA kit.

The formononetin and calycosin-7-O-β-D-glucopyranoside content were determined by high-performance liquid chromatography (HPLC) [35]. The dried root samples were ground into powder and passed through a 40-mesh sieve. A 1.0 g sample was extracted in methanol (50 mL) for 180 min in an ultrasonic bath. The weight of the extract solution was made up with methanol and the residue was removed by filtration. The extract solution was concentrated by a RE-52AA pressure rotary evaporator (Automatic Science, Shanghai, China), and dissolved in 5 mL methanol. The extract solution was filtered through a filter (0.22 μm) and transferred to a 2 mL autosampler vial. The filtrate (10 µL) was subjected to separation by HPLC using a reverse-phase C^18^ symmetry column (4.6 mm × 250 mm, pore size: 5 µm; Waters Corp., Milford, MA, USA). The mobile phase consisted of acetonitrile (A) and 0.2% formic acid (B). The separation was conducted in the gradient elution mode (0–20 min, 20%→40% A; 20–30 min, 40% A) at 30 °C with a flow rate of 1.0 mL/min. The eluted compounds were detected spectrophotometrically at 260 nm using a 2998 PDA photodiode array detector. The standard of formononetin and calycosin-7-O-β-D-glucopyranoside was purchased from the China National Institutes for Food and Drug Control.

### 2.6. Quantification of DSE Root Colonization

The fungal structures in the roots were observed to assess whether the roots of *A. mongholicus* were colonized by DSE [36]. Then, 0.5 cm fresh root segments were cleared in potassium hydroxide (10%, *w/v*) at 100 °C for 1 h and stained in acid fuchsin (0.5%, *w/v*) at 90 °C for 20 min. Thirty root segments of each sample were randomly selected to be observed by microscopy at 20× and 40× magnification.

### 2.7. Quantification of the N and P Contents of Plants

The 0.2 g dried plant samples were weighed for an elemental nitrogen and phosphorus analysis. Plant total phosphorus (TP) and nitrogen (TN) were measured by digestion with H_2_SO_4_–H_2_O_2_ and a whole-element autoanalyzer, respectively [37].

### 2.8. Soil Physicochemical Properties

Soil organic carbon (SOC) was measured by the scorch mass method [38], and soil available phosphorus (SAP) was measured by the sodium bicarbonate leaching–molybdenum antimony anticolorimetric method [39]. Alkaline phosphatase (ALP) activity was measured using p-nitrophenyl phosphate (PNPP) as a substrate [40]. Urease activity was determined using Kandeler and Gerber’s method based on the colorimetric determination of ammonium [41]. Soil ammonium (NH_4_^+^-N) and nitrate (NO_3_^−^-N) were measured by extraction from the soil with a 0.01 mol liter1 KCl 2 solution (1:100, wt/vol) and determination with a flow-injection autoanalyzer [42].

### 2.9. Statistical Analyses

Two-way analysis of variance (ANOVA) was used to assess the effects of DSE, *Trichoderma*, and their interactions on the measured parameters. Three-way variance analysis (ANOVA) was performed to examine the effects of DSE, *Trichoderma*, water, and their interactions on the plant growth parameters, antioxidant parameters, and active ingredient and soil nutrient contents. The correlations among the different variables were analyzed using the Pearson coefficient. Tukey’s tests and *t*-tests were used to compare the mean values to determine significant differences (*p* < 0.05). SPSS 21.0 software was used in the above analyses. Variation partitioning analysis (VPA) was used to analyze the interaction effects of DSE, *Trichoderma*, soil nutrients, and water on plant growth with the RStudio package “vegan”.

## 3. Results

### 3.1. DSE Colonization and Re-Isolation in Roots

After harvesting, DSE hyphae and microsclerotia structures were observed in all stained root segments (DSE inoculation, DSE + Trichoderma inoculation) of A. mongholicus under WW and DS treatment (Figure S3). No DSE structures were observed in non-inoculated plants and single-inoculated plants with Trichoderma strains.

Under WW conditions, co-inoculation with Trichoderma significantly increased DSE hyphal colonization (Figure S4a). Plants inoculated with L had significantly decreased P microsclerotia colonization (Figure S4b). Under DS conditions, L inoculation had significantly increased DSE hyphal and total colonization (Figure S4a,c). Inoculation with A significantly increased P hyphal colonization and total colonization (Figure S4). The DSE microsclerotia colonization of plants inoculated with L was higher, while the microsclerotia colonization of plants inoculated with A was lower than that of plants inoculated with M alone (Figure S4b).

### 3.2. Plant Growth Parameters

**Figure 1:**
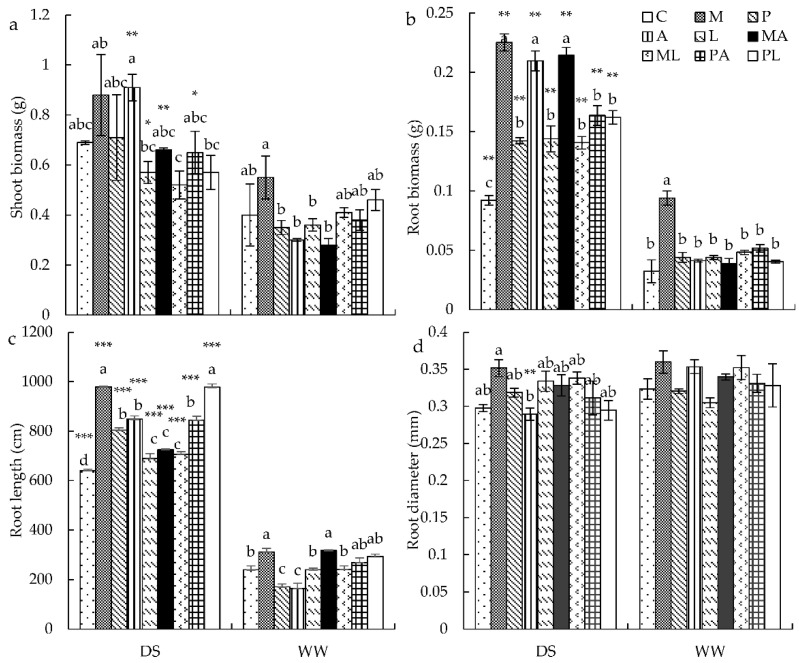
The effects of DSE inoculation, *Trichoderma* inoculation, and water conditions on the shoot biomass (**a**), root biomass (**b**), root length (**c**), and root diameter (**d**) of *Astragalus mongholicus*. The error bars represent the standard error of the mean. Different letters above the error bars indicate a significant difference at *p* < 0.05 by Tukey’s test. * means a significant difference between DS and WW. * *p* < 0.05, ** *p* < 0.01 and *** *p* < 0.001. The estimated means were presented when interactions were not significant. DS, drought stress conditions; WW, well-watered conditions. C indicates non-inoculated control. M, P, A, L, MA, ML, PA, and PL indicate plants inoculated with *Macrophomina pseudophaseolina*; *Paraphoma radicina*; *Trichoderma afroharzianum*; *Trichoderma longibrachiatum*; *M. seudophaseolina* + *T. froharzianum*; *M. seudophaseolina* + *T. longibrachiatum*; *P. radicina* + *T. afroharzianum*; *P. radicina* + *T. longibrachiatum*.

Under WW conditions, shoot biomass and root biomass improved by 37.5% and 191.02%, respectively, after *M* inoculation compared to control plants (Figure 1a,b). Under DS conditions, inoculation with *M* and *A* increased shoot biomass by 27.54% and 31.88% as compared to the control plants (Figure 1a). Single and dual inoculation significantly increased root biomass compared to nonincubated plants (Figure 1b). In particular, *M* inoculation, *A* inoculation, and *MA* inoculation led to higher root biomass compared to other inoculation treatments.

Under WW conditions, *M* inoculation significantly increased root length (+29.69%), while *PA* inoculation significantly increased root length compared to single-inoculated plants (Figure 1c). Inoculation treatments had no effects on root diameter under DS and WW conditions (Figure 1d). Drought significantly enhanced root length compared to plants grown in WW conditions. Under DS conditions, all inoculations led to significantly improved root length compared to non-inoculated plants (Figure 1c). The root length in plants inoculated with *PL* grew larger than that in plants inoculated with *P* or *L* separately.

### 3.3. Osmotic Materials and Antioxidant Enzyme Activities in Leaves

**Figure 2:**
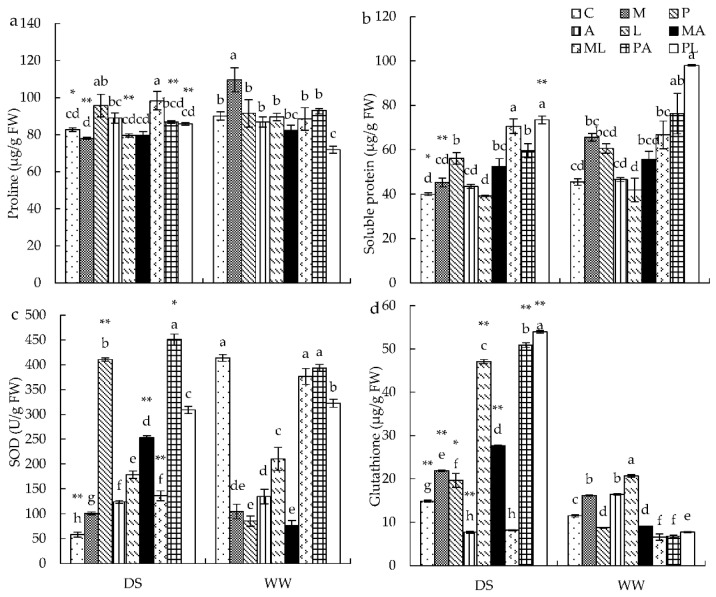
The effects of DSE inoculation, *Trichoderma* inoculation, and water conditions on the proline content (**a**), soluble protein content (**b**), SOD activity (**c**), and glutathione content (**d**) of *Astragalus mongholicus*. The error bars represent the standard error of the mean. Different letters above the error bars indicate a significant difference at *p* < 0.05 by Tukey’s test. * means a significant difference between DS and WW. * *p* < 0.05, ** *p* < 0.01. DS, drought stress conditions; WW, well-watered conditions. C indicates non-inoculated control. M, P, A, L, MA, ML, PA, and PL indicate plants inoculated with *Macrophomina pseudophaseolina*; *Paraphoma radicina*; *Trichoderma afroharzianum*; *Trichoderma longibrachiatum*; *M. seudophaseolina + T. froharzianum; M. seudophaseolina + T. longibrachiatum; P. radicina + T. afroharzianum; P. radicina + T. longibrachiatum*.

GSH content and SOD activity were significantly influenced by water, DSE, *Trichoderma*, and their interaction. Soluble protein content was significantly affected by water, DSE, *Trichoderma*, and co-inoculation of DSE and *Trichoderma*. Proline content was significantly impacted by *Trichoderma* inoculation, interaction of water and DSE, and interaction of DSE and *Trichoderma* (Table S1). Under WW conditions, *M* inoculation significantly increased proline content, *M* and *P* inoculation increased soluble protein content, DSE and *Trichoderma* inoculation significantly decreased SOD activity, and *M*, *A*, and *L* inoculation significantly increased GSH content compared with control plants (Figure 2). *PL* and *PA* had higher soluble protein content, while *ML*, *PA*, and *PL* had significantly higher SOD activity than in those inoculated with a single strain (Figure 2). Under DS conditions, *P* inoculation significantly increased proline content (+15.77%), and plants inoculated with *ML* had higher proline content than those inoculated with *M* and *L* (Figure 2a). Inoculation with DSE and *Trichoderma* (except for *L*) enhanced soluble protein content, while the soluble protein content inoculated with *MA*, *ML*, and *PL* was higher than that of plants inoculated with single DSE and *Trichoderma* (Figure 2b). SOD activity was significantly enhanced by all inoculations; SOD activity after inoculating with *MA* and *PA* was higher than that inoculated with a single strain (Figure 2c). GSH content was significantly increased by *M*, *P*, and *A* inoculation compared with control plants. Plants inoculated with *MA*, *PA*, and *PL* had higher GSH content than those inoculated with single DSE and *Trichoderma* (Figure 2d).

### 3.4. Antioxidant Parameters and IAA Contents in Roots

**Figure 3:**
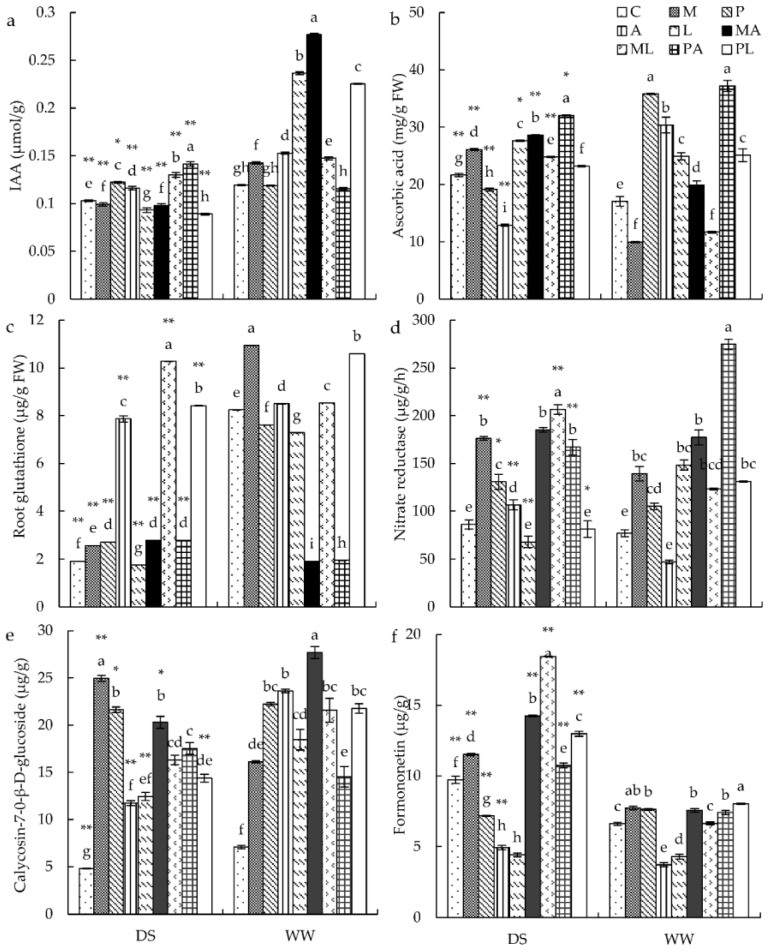
The effects of DSE inoculation, *Trichoderma* inoculation, and water conditions on the IAA content (**a**), ascorbic acid content (**b**), root glutathione content (**c**), nitrate reductase activity (**d**), calycosin-7-O-β-D-glucoside content (**e**), and formononetin content (**f**) of *Astragalus mongholicus*. The error bars represent the standard error of the mean. Different letters above the error bars indicate a significant difference at *p* < 0.05 by Tukey’s test. * means a significant difference between DS and WW. * *p* < 0.05, ** *p* < 0.01. DS, drought stress conditions; WW, well-watered conditions. C indicates non-inoculated control. M, P, A, L, MA, ML, PA, and PL indicate plants inoculated with *Macrophomina pseudophaseolina*; *Paraphoma radicina*; *Trichoderma afroharzianum*; *Trichoderma longibrachiatum*; *M. seudophaseolina + T. froharzianum; M. seudophaseolina + T. longibrachiatum*; *P. radicina + T. afroharzianum; P. radicina + T. longibrachiatum*.

**Figure 4:**
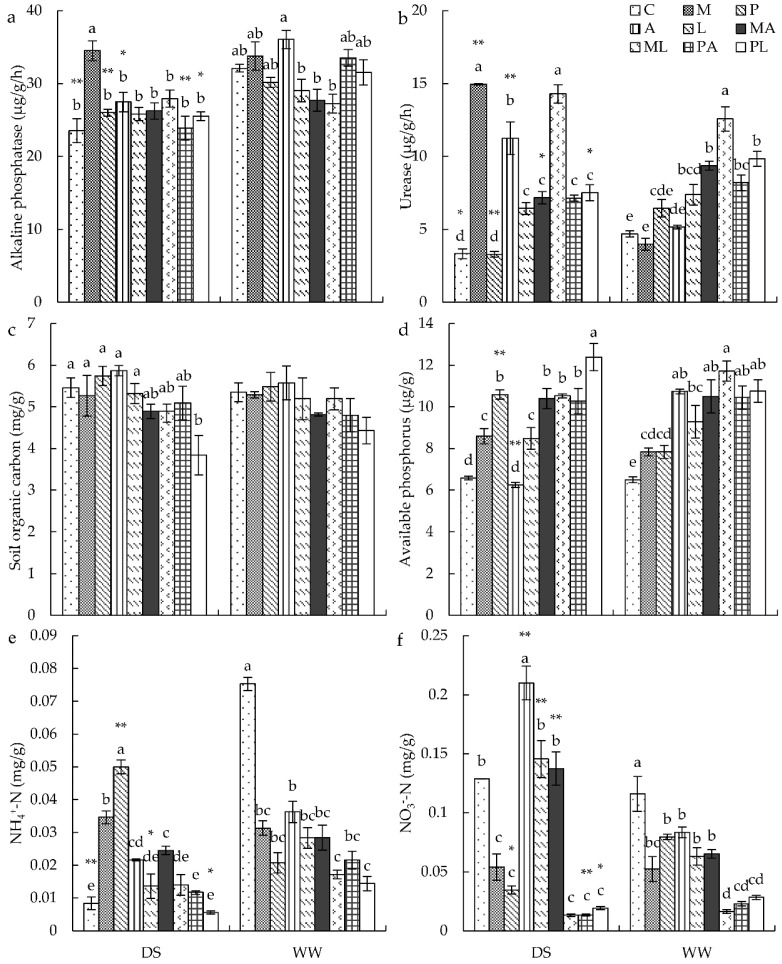
The effects of DSE inoculation, *Trichoderma* inoculation, and water conditions on soil alkaline phosphatase (**a**), soil urease (**b**), soil organic carbon (**c**), soil available phosphorus (**d**), soil ammonia nitrogen (**e**), and soil nitrate nitrogen (**f**). The error bars represent the standard error of the mean. Different letters above the error bars indicate a significant difference at *p* < 0.05 by Tukey’s test. * means a significant difference between DS and WW. * *p* < 0.05, ** *p* < 0.01. The estimated means were presented when interactions were not significant. DS, drought stress conditions; WW, well-watered conditions. C indicates non-inoculated control. M, P, A, L, MA, ML, PA, and PL indicate plants inoculated with *Macrophomina pseudophaseolina*; *Paraphoma radicina*; *Trichoderma afroharzianum*; *Trichoderma longibrachiatum*; *M. seudophaseolina + T. froharzianum; M. seudophaseolina + T. longibrachiatum; P. radicina + T. afroharzianum; P. radicina + T. longibrachiatum*.

GSH content was significantly influenced by all treatments and their interaction. NR activity and ASA content were significantly affected by all inoculations and their interaction (Table S1). Under WW conditions, *M*, *A*, and *L* inoculation significantly increased IAA content, while *MA* inoculation significantly improved IAA content compared with plants inoculated with *M* or *A* alone (Figure 3a). ASA contents of single-inoculated plants (except for *M* inoculation) were significantly increased (Figure 3b). GSH content was significantly improved in *A. mongholicus* by *M* and *A* inoculation, while *PL* inoculation significantly increased GSH content compared with single-inoculated plants (Figure 3c). All inoculated plants (except for *A* inoculation) significantly enhanced NR activity compared with the control plants, while *PA* inoculation had higher NR activity than *P* and *A* inoculation (Figure 3d). Under DS conditions, inoculation with *P* and *A* significantly increased IAA content, while inoculation with *M* and *L* had significantly decreased IAA content compared to control plants. Plants inoculated with *ML* and *PA* had higher IAA content than those inoculated with *M*, *P*, and *L* (Figure 3a). ASA content was significantly enhanced after inoculation with *M* and *L*, and significantly reduced after inoculation with *P* and *A* compared with the control plants. The ASA content of plants inoculated with *MA* and *PA* was larger than in those inoculated with *M*, *P*, and *A* (Figure 3b). The GSH contents of inoculated plants (except for *L* inoculum) were significantly enhanced compared with the control plants, and the GSH contents in plants inoculated with *ML* and *PL* were higher than in plants inoculated with a single strain (Figure 3c). The NR activity of plants inoculated by *M*, *P*, and *A* was significantly enhanced compared with the control plants (Figure 4d). The NR activity of plants inoculated with *ML* and *PA* was higher than that of plants inoculated with *M*, *P*, and *A* (Figure 3d).

### 3.5. Active Ingredient Contents in the Roots

Calycosin-7-O-glucoside and formononetin contents were significantly impacted by all tested treatments and their interaction (Table S1). Under WW conditions, all inoculated plants had significantly increased calycosin-7-O-glucoside contents compared with the control plants (Figure 3e). The formononetin content of plants with *M* and *P* were significantly enhanced compared with the control plants (Figure 3f). Inoculation with *MA* and *PL* led to a higher calycosin-7-O-glucoside and formononetin content, respectively, than single inoculation. Under DS conditions, all single-inoculated plants had significantly increased calycosin-7-O-glucoside contents compared with the control plants (Figure 3e). The formononetin content of plants with *M* inoculation was significantly increased compared to control plants. The content of formononetin in co-inoculated plants was higher than that in single-inoculated plants (Figure 3f).

### 3.6. N and P Content in the Shoots and Roots 

Plant TN content was significantly influenced by all treatments and their interactions, and plant TP content was significantly influenced by all treatments, except for water condition (Table S1). Under WW treatment, plant TN and TP contents were significantly increased by *P* and *L* inoculation, while *A* inoculation only significantly enhanced plant TP content compared with control plants (Figure S4). Under DS treatment, inoculation with *MA* and *ML* had an activity effect on plant TN content compared with *M*, *A*, and *L* inoculation (Figure S4a). Inoculation with *M* and *L* significantly increased the plant TP content compared to control plants (Figure S4b).

### 3.7. Soil Factors

Soil urease activity and NO_3_^−^-N content were significantly influenced by all treatments and their interactions, while water condition had no effect on NH_4_^+^-N content. SOC and SAP contents were significantly impacted by DSE and *Trichoderma* inoculation. ALP activity was significantly affected by water condition, *Trichoderma* inoculation, the interaction between water and DSE, and the interaction between DSE and *Trichoderma* (Table S1).

Under WW conditions, *L* inoculation significantly improved soil urease activity, while *MA* and *ML* inoculation increased soil urease activity more than single inoculation.

All single inoculations significantly enhanced SAP content but decreased NH_4_^+^-N content and NO_3_^−^-N content (Figure 4). Under DS conditions, inoculation with *M* significantly enhanced ALP activity (Figure 4a). Single inoculation (except for *P* inoculation) significantly increased soil urease activity (Figure 4b). Co-inoculation of *PL* significantly decreased SOC content (Figure 4c). SAP content was significantly improved by all single inoculations except for *A*, and *MA*, *ML*, and *PL* inoculation led to higher SAP content than single DSE or *Trichoderma* inoculation (Figure 4d). Inoculation with *M*, *P*, and *A* significantly increased NH_4_^+^-N content, but it was decreased by *PA* inoculation (Figure 4e). NO_3_^−^-N content was significantly improved by *A* inoculation, but was decreased by *M* and *P* inoculation (Figure 4f).

### 3.8. Correlation Analyses of Growth Parameters and Chemical Constituents

Pearson correlation analyses showed a correlation between soil nutrient elements and growth parameters of *A. mongholicus* inoculated with DSE, *Trichoderma*, as well as DSE–*Trichoderma* under DS conditions (Table 1 and Tables S2–S4). Under DSE inoculation, ALP and urease activity were positively correlated with root biomass, root length, formononetin content, and NR activity. SAP and NH_4_^+^-N contents were positively correlated with Calycosin-7-O-glucoside and IAA contents. NO_3_^−^-N content was negatively correlated with root biomass, root length, calycosin-7-O-glucoside content, and NR activity. Under *Trichoderma* inoculation, shoot biomass, root biomass, and IAA content were positively correlated with urease activity, NH_4_^+^-N, and NO_3_^−^-N contents. Root length and calycosin-7-O-glucoside content were positively correlated with urease activity and NH_4_^+^-N content. However, SAP content was negatively correlated with shoot biomass and IAA content. Under co-inoculation, SAP content was positively correlated with root biomass, root length, and calycosin-7-O-glucoside content. NH_4_^+^-N content was positively correlated with root biomass, calycosin-7-O-glucoside content, and NR activity. Urease activity was positively correlated with calycosin-7-O-glucoside content, formononetin content, IAA content, and NR activity. Nevertheless, NO_3_^−^-N content was negatively correlated with root length and IAA content.

### 3.9. Variation Partitioning Analysis of Growth Parameters and Chemical Constituents

**Figure 5:**
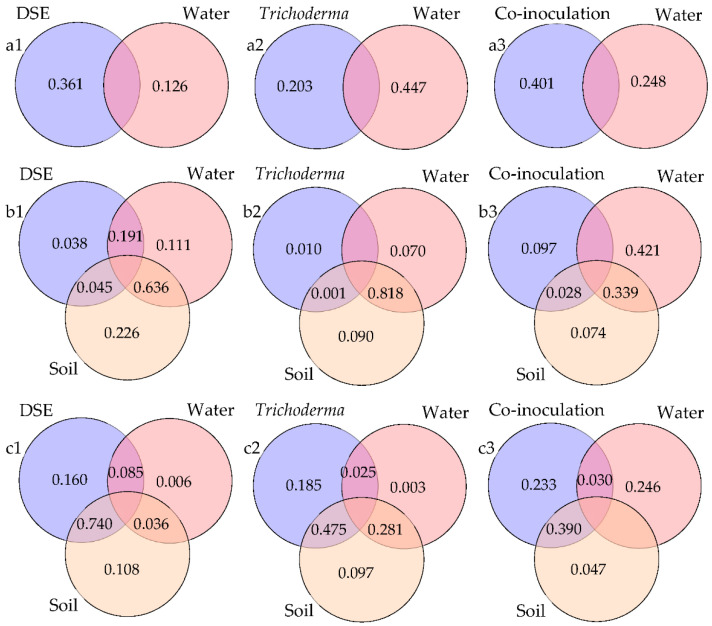
The variation partitioning analysis (VPA) of soil factors (**a1**–**a3**), growth indicators (**b1**–**b3**), and active ingredient contents (**c1**–**c3**) of *Astragalus mongholicus*. (**a1**): VPA of DSE inoculation and water conditions on soil factors; (**a2**): VPA of *Trichoderma* inoculation and water conditions on soil factors; (**a3**): VPA of co-inoculation and water conditions on soil factors. (**b1**): VPA of DSE inoculation, water conditions, and soil factors on growth and physiological parameters of *A. mongholicus*; (**b2**): VPA of *Trichoderma* inoculation, water conditions, and soil factors on growth and physiological parameters of *A. mongholicus*; (**b3**): VPA of co-inoculation, water conditions, and soil factors on growth and physiological parameters of *A. mongholicus*. (**c1**): VPA of DSE inoculation, water conditions, and soil factors on active ingredient contents of *A. mongholicus*; (**c2**): VPA of *Trichoderma* inoculation, water conditions, and soil factors on active ingredient contents of *A. mongholicus*; (**c3**): VPA of co-inoculation, water conditions, and soil factors on active ingredient contents of *A. mongholicus*.

VPA was used to quantify the association between DSE species, *Trichoderma* species, water condition, soil nutrients, and plant growth (Figure 5). Co-inoculation explained 40.1% of soil factors, which was higher than that of DSE inoculation (36.1%) and *Trichoderma* inoculation (20.3%), indicating that co-inoculation may make a greater contribution to soil nutrition (Figure 5a). The effect of water condition on soil factors was smaller than that of DSE inoculation and co-inoculation (Figure 5a1,a2). Co-inoculation (9.7%) explained more plant growth and physiological parameters than DSE inoculation (3.8%) and *Trichoderma* inoculation (1%) (Figure 5b). The amount of medicinal ingredients explained by co-inoculation (23.3%) was greater than that by DSE inoculation (16%) and *Trichoderma* inoculation (18.5%) (Figure 5c). For the contents of active ingredients, the effect of interaction between soil and inoculation was greater than that of soil or inoculation alone (Figure 5c).

## 4. Discussion

The interaction between microorganisms and plants in nutrient absorption, growth, and defense of the system is an integral part of the ecosystem [43]. Microorganisms are considered to be natural partners of plant defense mechanisms under adverse conditions [44]. Although many studies showed that DSE and *Trichoderma* can promote the growth of plants, few studies have been performed on the growth-promoting mechanisms of plants co-inoculated with DSE and *Trichoderma* under drought stress. In this study, typical DSE hyphae and microsclerotia were found in all treated root samples, indicating that DSE could effectively colonize the roots of *A. mongholicus*, even under drought conditions. Inoculation of *T. longibrachiatum* and *T. afroharzianum* promoted the colonization of *M. pseudophaseolina* and *P. radicina*, indicating that *Trichoderma* and DSE could coexist in the roots of *A. mongholicus*.

In this study, the root biomass and root length of *A. mongholicus* under drought stress were better than those under well-watered conditions. It has been proven that allocating greater biomass to roots is a key mechanism to enhance drought tolerance in plants [45]. Under drought conditions, DSE and *Trichoderma* inoculation had no significant effects on shoot biomass, but DSE and *Trichoderma* inoculation made significant contributions to the root biomass and root length of *A. mongholicus*. Li et al. [46] found that DSE inoculation can regulate the root biomass and root architecture of *Hedysarum scoparium* to improve the performance of plants under drought conditions. *T. harzianum* significantly increased root growth and development in maize and several other crop plants [47]. The application of DSE or *Trichoderma* led to the development of deep and extensive roots to penetrate farther into the soil, which helped to absorb deep-seated water and nutrients to enhance drought tolerance in plants [48,49]. Under drought stress, the effect of *P. radicina* + *T. longibrachiatum* on root length was better than that of *P. radicina* and *T. longibrachiatum* alone. He et al. [28] found that three DSE strains inoculated with *Trichoderma viride* at different concentrations in *Glycyrrhiza uralensis* and dual inoculation can enhance the performance of *G. uralensis* by promoting root development. Wang et al. [50] found that the co-inoculation of *Funnelliformis mosseae* and *Sinorhizobium medicae* improved the nutrient absorption and growth of alfalfa, which could be an effective way to improve productivity. In addition, DSE and *Trichoderma* inoculation significantly enhanced calycosin-7-O-glucoside content, and the effect of co-inoculation on the content of active ingredients was greater than that of single inoculation. Li et al. [51] found that DSE inoculation enhanced the epigoitrin content of *Isatis tinctoria* under drought. The withanolide A content of *T. viride*-treated plants increased by co-inoculation of *T. viride*, and *Sarocladium kiliense* modulated the expression of the withanolide biosynthetic pathway genes of *Withania somnifera* [52]. Thus, DSE and *Trichoderma* inoculation can be used to stimulate the production of more active ingredients in medicinal plants.

Drought stress causes excessive production of reactive oxygen species (ROS) in plants, leading to oxidative stress that damages plant cells and ultimately reduces plant growth and development [53]. When stressed, plants produce effective enzymes such as SOD to remove ROS and protect cells from oxidative damage [54]. Our results showed that both DSE and *Trichoderma* inoculation had positive effects on SOD activity, and *M. pseudophaseolina* + *T. afroharzianum* and *P. radicina* + *T. longibrachiatum* inoculation had higher SOD activity than single inoculation. Li et al. [51] revealed that inoculation with DSE can enhance SOD activity under drought stress. Inoculation with *Trichoderma* resulted in overexpression of SOD metabolic pathway genes and significantly increased SOD activity in drought-stressed rice plants as compared to control plants [55]. Singh et al. [56] found that increasing SOD activity can improve ROS scavenging ability in rice plants with co-inoculation of *Trichoderma asperellum* and *Pseudomonas fluorescens*. These results indicated that inoculation with DSE and *Trichoderma* can improve antioxidant enzyme activity and alleviate the oxidative damage induced by drought. In response to osmotic stress caused by drought, osmotic regulation is also an important mechanism for plants to regulate water potential to adapt to drought stress [57]. Soluble proteins, as compatible osmotic regulators, can promote osmotic regulation and plant tolerance to dehydration. Although drought resulted in decreased protein synthase activity and protein content in plants, inoculation with DSE and *Trichoderma* significantly improved the soluble protein content in this study, and the effect of co-inoculation was higher than that of individual inoculation. These results suggest that DSE and *Trichoderma* inoculation can promote the production of soluble proteins to alleviate the negative effects of drought [58,59]. Proline plays an important role in osmotic regulation, protein property stability, and cell structure stability under drought stress [4]. Valli and Muthukumar [60] also found that DSE inoculation could significantly enhance proline accumulation in tomato shoots. Rawat et al. [61] indicated that *T. harzianum* inoculation increased proline content to reduce the oxidative damage caused by salt stress. Rezaei-Chiyaneh et al. [62] found that co-inoculated plants under severe water stress produced the highest proline contents in ajowan leaves. Thus, DSE and *Trichoderma* inoculation could promote the biosynthetic activity of proline to regulate osmotic pressure under water deficiency. GSH is an important antioxidant composed of glutamic acid, cysteine, and glycine, and the sulfhydryl structure contained in it can combine with reactive oxygen species, thereby reducing the oxidative damage of cells [63]. ASA is a water-soluble antioxidant organic small molecule widely present in plants. In order to maintain redox balance in stress, the ASA–GSH cycle, as the main nonenzymatic antioxidant to maintain redox dynamic balance in cells, is the main defense system against ROS damage in cells [64]. Sadeghi et al. [65] found that inoculation with endophytic fungi significantly increased GSH and ASA content in citrus under drought to induce drought tolerance in the host. In the present study, *A. mongholicus* inoculated with DSE and *Trichoderma* under drought stress obtained a large amount of GSH and ASA, which adjusted the redox state and prevented cell damage. Whereas inoculation with *T. afroharzianum* and *M. pseudophaseolina* + *T. longibrachiatum* decreased the GSH content in leaves, inoculation with *P. radicina* and *T. afroharzianum* decreased the ASA content in roots. Mastouri et al. [66] also discovered similar results in maize inoculated with *Trichoderma* isolate T-22. This may be due to the conversion of reduced GSH and ASA to a more oxidized form with the increase of ROS, which directly or indirectly removes ROS [64,65]. These results show that DSE and *Trichoderma* inoculation increased enzymatic and nonenzymatic antioxidant reactions as well as metabolites induced by inoculation, which acted as agents of quenching and contributed strongly to alleviating drought in plants.

Plant hormones play an important role in plant growth and development and in the interaction between plants and endophytes [67]. IAA, the most abundant auxin in nature, plays a central role in beneficial root–microbial interactions and is generally thought to be involved in balancing growth and defense responses, coordinating many key processes in plant growth [68]. Although drought reduced IAA levels, IAA content was enhanced by inoculating *P. radicina* and *T. afroharzianum*. Additionally, the IAA content of plants co-inoculated with *M. pseudophaseolina* + *T. longibrachiatum* and *P. radicina* + *T. afroharzianum* was higher than that of individual inoculation under drought conditions. It was found that the key enzymes encoding IAA synthesis were significantly upregulated under DSE treatment [69], and DSE colonization promoted auxin production in plants [70]. Qiang et al. [71] also found that wheat plants inoculated with an IAA-producing endophytic fungus, *Alternaria alternata* LQ1230, could improve wheat growth and enhance drought tolerance. *Trichoderma* strains could also increase IAA secretion in response to drought, showing the ability to protect plants [14,15,58]. Coculture of *Arabidopsis* seedlings with *Trichoderma virens* or *Trichoderma atroviride* showed characteristic auxin-induced phenotypes, including increased biomass production and stimulated lateral root development [72]. In addition, IAA concentration in tobacco plants was enhanced by co-inoculating *Glomus versiforme* and *Bacillus methylotrophicus*, and the IAA concentration in co-inoculated plants was higher than that in single-inoculated plants [73]. The interaction between beneficial microorganisms and plants can regulate the hormone metabolism of the associated plants to induce root growth and development and promote nutrient absorption by host plants, thus improving the drought tolerance of plants [72,74].

The results of a variance decomposition showed that DSE and co-inoculation explained more for soil factors than water treatments, so the addition of exogenous microorganisms inevitably affects soil properties. NO_3_^−^-N and NH_4_^+^-N are the main inorganic nitrogen sources that plants can absorb and utilize [75]. NR (nitrate reductase) is a key enzyme in the nitrate assimilation pathway of higher plants, which can transform nitric acid to ammonia and improve the absorption of N by plants [74]. Therefore, its activity can be used to evaluate the ability of plants to absorb and assimilate N [76]. Urease can convert urea into ammonia and increase the content of N in soil [77]. In this study, inoculation with DSE and *Trichoderma* could affect NR, urease, NH_4_^+^-N, and NO_3_^−^-N. Upson et al. [78] found that DSE are able to mineralize peptides and amino acids in the rhizosphere, making N more freely available to roots. Wu et al. [79] indicated that colonization by DSE S16 significantly promoted the activity of NR and some genes involved in nitrate regulation in sweet cherry. Some existing research found that DSE hyphal infection and *Trichoderma* treatment were positively correlated with soil urease activity [80,81]. Thus, DSE and *Trichoderma* are involved in regulating the uptake of nitrate by plants, and sufficient N supply may contribute to osmoregulation and ROS homeostasis in plants, making plants better adapted to drought [82]. In addition, the content of available P increased, which may be due to DSE and *Trichoderma* dissolving insoluble phosphate in the soil and using it as a biofertilizer to aid plant growth. Studies have shown that the organic acids produced by DSE cause the dissolution of phosphates, helping to enhance P supply to plants [83]. *Trichoderma* M14 had the ability to solubilize unavailable P for plant growth [84]. *T. Asperellum* produced phosphatases and dissolved inorganic or organic phosphates [85]. The content of available P in soils inoculated with *M. pseudophaseolina* + *T. afroharzianum*, *M. pseudophaseolina* + *T. longibrachiatum*, and *P. radicina* + *T. longibrachiatum* was higher than that of single inoculation, so dual inoculation with compatible strains could play a synergistic role in improving plant mineral nutrient absorption. The above results indicate that DSE and *Trichoderma* inoculation can increase soil enzyme activities to accelerate soil fertility. At the same time, the organic N and insoluble P in the soil were mineralized, the content of available N and available P was increased to improve the utilization rate of macro and trace elements of plants, and it increased the interaction between plants and soil.

The VPA showed that soil nutrients had a greater impact on plant growth under DSE treatment. Correlation analysis showed that DSE inoculation increased the activity of NR in roots and soil enzymes to promote the absorption of nitrate N by plants to promote the growth of plant roots and the content of active ingredients. Deng et al. [86] demonstrated that the biosynthesis of flavonoids is strongly influenced by N status, and N availability alters flavonoid accumulation by affecting the internal carbon/nitrogen balance. Meanwhile, the enlarged plant roots could absorb more soil inorganic matter and form a good cycle. *Trichoderma* inoculation increased the activity of soil urease and accelerated the production of soil NH_4_^+^-N, which promoted the growth of plants. *Trichoderma* inoculation promotes plant uptake of available P to increase aboveground biomass and IAA content. It was found that IAA can upregulate genes encoding enzymes responsible for the synthesis of carboxylates involved in P solubilization to increase soluble P content [87]. P mainly increased root growth by upregulating the positive regulation root growth gene, and then affected root nutrient absorption and plant growth [88]. Hence, P was related to the increased IAA content [89]. Co-inoculation promoted the mineralization of insoluble P and soil available P content, which eventually led to the utilization of available P by plant roots. Co-inoculation increased the NH_4_^+^-N content in the soil, resulting in an increase in plant root growth and the content of active ingredients. These results illustrate that, in addition to profound physiological changes in plants following DSE and *Trichoderma* inoculation, DSE and *Trichoderma* mediated responses to plant–soil feedback. Therefore, functional symbiosis is established by regulating plant drought resistance, improving soil nutrition, and promoting plant growth and other mechanisms.

## 5. Conclusions

In this study, we found that DSE can effectively colonize the roots of *A. mongholicus*, and *Trichoderma* promoted the colonization of DSE in the roots of *A. mongholicus*. DSE and *Trichoderma* inoculation significantly enhanced growth and active ingredient contents of *A. mongholicus* regardless of the water condition. Interestingly, under drought stress, *P. radicina* + *T. longibrachiatum* had larger root length and co-inoculation significantly enhanced the formononetin content of *A. mongholicus* compared to single inoculation. Additionally, *Trichoderma* relies on antioxidant enzymes, growth hormones, and a redox system to resist drought, while DSE strains have an additional osmotic regulation system in addition to the drought resistance function possessed by *Trichoderma*, and the effect of co-inoculation (especially *M*. *pseudophaseolina* + *T. longibrachiatum* and *P. radicina* + *T. afroharzianum*) on plant physiology was greater than that of single inoculation. DSE inoculation increased soil enzymes to promote nitrate nitrogen absorption by plants. *Trichoderma* inoculation increased soil urease activity and plant uptake of available phosphorus. Co-inoculation promoted the mineralization of insoluble phosphorus in soil. Our results supplement previous findings that endophytes can enhance the drought resistance of plants and emphasize the importance of using DSE and *Trichoderma* in medicinal plant cultivation under drought stress conditions. As *A. mongholicus* plays an important role in the cultivation of medicinal plants, the DSE–*Trichoderma*–*A. mongholicus* association should be further field-tested to determine its ability to suppress drought stress in dryland agriculture.

## Figures and Tables

**Table 1:** Correlation analysis of the growth indicators and soil properties of *Astragalus membranaceus* inoculated with DSE and *Trichoderma* under drought stress condition.

		SAP	ALP	U	NH_4_^+^-N	NO_3_^−^-N
DSE	Shoot biomass	0.008	0.341	0.358	−0.083	−0.023
	Root biomass	0.366	0.915 **	0.924 **	0.117	−0.643 *
	Root length	0.391	0.734 *	0.786 **	0.210	−0.639 *
	Calycosin-7-O-glucoside	0.808 **	0.664 *	0.542	0.656 *	−0.937 **
	Formononetin	−0.564	0.598 *	0.808 **	−0.769 **	0.276
	IAA	0.715 *	−0.444	−0.662 *	0.891 **	−0.470
	Nitrate reductase	0.471	0.842 **	0.843 **	0.240	−0.694 *
*Trichoderma*	Shoot biomass	−0.799 **	0.362	0.717 *	0.592 *	0.729 *
	Root biomass	−0.084	0.498	0.950 **	0.887 **	0.842 **
	Root length	−0.304	0.307	0.761 **	0.603 *	0.549
	Calycosin-7-O-glucoside	0.422	0.465	0.703 *	0.600 *	0.528
	Formononetin	−0.404	−0.498	−0.702 *	−0.610 *	−0.543
	IAA	−0.683 *	0.269	0.706 *	0.633 *	0.716 *
	Nitrate reductase	−0.658 *	0.006	0.544	0.558	0.708 *
Co-inoculation	Shoot biomass	−0.284	−0.074	−0.546 *	0.019	0.438
	Root biomass	0.612 **	0.260	0.201	0.656 **	0.026
	Root length	0.567 *	−0.103	0.036	−0.251	−0.486 *
	Calycosin-7-O-glucoside	0.646 **	0.379	0.504 *	0.604 **	−0.288
	Formononetin	0.416	0.623 **	0.913 **	0.343	−0.313
	IAA	−0.041	0.020	0.454 *	0.036	−0.535 *
	Nitrate reductase	0.144	0.349	0.677 **	0.658 **	−0.140

IAA = indole-3-acetic acid; SAP = soil available phosphorus; ALP = soil alkaline phosphatase; U = urease; NH_4_^+^-N = soil ammonia nitrogen; NO_3_^−^-N = soil nitrate nitrogen. * and ** indicate significance at *p* ≤ 0.05 and *p* ≤ 0.01, respectively.

## Data Availability

The datasets used and/or analyzed during the current study are available from the corresponding author upon reasonable request.

## Supplementary Materials

The following supporting information can be downloaded at: https://www.mdpi.com/article/10.3390/jof8090920/s1, Figure S1: Colonies and microscopic morphology of DSE and *Trichoderma*. A(a)-D(d) indicate *Macrophomina pseudophaseolina* (A, a); *Paraphoma radicina* (B, b); *Trichoderma afroharzianum* (C, c); *Trichoderma longibrachiatum* (D, d). Scale bars = 50 μm.; Figure S2: Maximum likelihood tree based on rDNA ITS region sequences of DSE and *Trichoderma* (**a**) and tef1 sequences of *Trichoderma* (**b**).; Figure S3: Colonization of dark septate endophytes (DSE) in the roots of *Astragalus mongholicus* four months after inoculation. W: Well-watered; D: drought stress; M: *Macrophomina pseudophaseolina*; P: *Paraphoma* *radicina*; MA: *M pseudophaseolina + Trichoderma afroharzianum*; ML: *M pseudophaseolina* + *Trichoderma longibrachiatum*; PA: *P radicina* + *T* *afroharzianum*; PL: *P radicina* + *T* *longibrachiatum*. Hy indicates DSE hyphae, Mi indicates DSE microsclerotia. Scale bars = 50 μm.; Figure S4: Hyphae colonization rate (**a**), microsclerotia colonization rate (**b**), and total colonization rate (**c**) of dark septate endophytes (DSE) in the roots of *Astragalus mongholicus* four months after inoculation. DS: drought stress; WW: Well-watered; M: *Macrophomina pseudophaseolina*; P: *Paraphoma radicina*; MA: *M pseudophaseolina + Trichoderma afroharzianum*; ML: *M pseudophaseolina* + *Trichoderma longibrachiatum*; PA: *P radicina* + *T* *afroharzianum*; PL: *P radicina* + *T* *longibrachiatum*.; Figure S5: The effects of DSE inoculation, *Trichoderma* inoculation, and water conditions on total nitrogen (**a**) and total phosphorus (**b**) of *Astragalus mongholicus*. The error bars represent the standard error of the mean. Different letters above the error bars indicate a significant difference at *p* < 0.05 by Tukey’s test. DS, drought stress conditions; WW, well-watered conditions. C indicates non-inoculated control. M, P, A, L, MA, ML, PA, and PL indicate plants inoculated with *Macrophomina pseudophaseolina*; *Paraphoma radicina*; *Trichoderma afroharzianum*; *Trichoderma longibrachiatum*; *M. seudophaseolina + T. froharzianum; M. seudophaseolina + T longibrachiatum; P radicina + T afroharzianum; P radicina + T longibrachiatum.*; Table S1: Analysis of variance (ANOVA) for the effects of water condition, DSE inoculation, and Trichoderma inoculation on the growth of *Astragalus membranaceus*. GSH = glutathione; SOD = superoxide dismutase; NR = nitrate reductase; ASA = ascorbic acid; MDA = malondialdehyde; IAA = indole-3-acetic acid; SOC = soil organic carbon; SAP = soil available phosphorus; ALP = soil alkaline phosphatase; U = urease; NH_4_^+^-N = soil ammonia nitrogen; NO_3_^−^-N = soil nitrate nitrogen. * and ** indicate significance at *p* ≤ 0.05 and *p* ≤ 0.01, respectively. Table S2: Correlation analysis of the growth indicators and soil properties of *Astragalus membranaceus* inoculated with DSE under drought stress condition. SB = shoot biomass; RB = root biomass; RL = root length; C = calycosin-7-O-β-D-glucoside; F = formononetin; IAA = indole-3-acetic acid; NR = root nitrate reductase; SAP = soil available phosphorus; ALP = soil alkaline phosphatase; U = urease; NH_4_^+^-N = soil ammonia nitrogen; NO_3_^−^-N = soil nitrate nitrogen. * and ** indicate significance at *p* ≤ 0.05 and *p* ≤ 0.01, respectively.; Table S3: Correlation analysis of the growth indicators and soil properties of *A. membranaceus* inoculated with *Trichoderma* under drought stress condition. SB = shoot biomass; RB = root biomass; RL = root length; C = calycosin-7-O-β-D-glucoside; F = formononetin; IAA = indole-3-acetic acid; NR = root nitrate reductase; SAP = soil available phosphorus; ALP = soil alkaline phosphatase; U = urease; NH_4_^+^-N = soil ammonia nitrogen; NO_3_^−^-N = soil nitrate nitrogen. * and ** indicate significance at *p* ≤ 0.05 and *p* ≤ 0.01, respectively; Table S4: Correlation analysis of the growth indicators and soil properties of *A. membranaceus* co-inoculated with DSE and *Trichoderma* under drought stress condition. SB = shoot biomass; RB = root biomass; RL = root length; C = calycosin-7-O-β-D-glucoside; F = formononetin; IAA = indole-3-acetic acid; NR = root nitrate reductase; SAP = soil available phosphorus; ALP = soil alkaline phosphatase; U = urease; NH_4_^+^-N = soil ammonia nitrogen; NO_3_^−^-N = soil nitrate nitrogen. * and ** indicate significance at *p* ≤ 0.05 and *p* ≤ 0.01, respectively.
