# Supplementary material for: Complementary Effects of Dark Septate Endophytes and Trichoderma Strains on Growth and Active Ingredient Accumulation of Astragalus mongholicus under Drought Stress
Source: J Fungi (Basel). 2022 Aug 30;8(9):920. doi: 10.3390/jof8090920 (PMC9506129; doi:10.3390/jof8090920)
Supplement: Supplementary file 1 [file jof-08-00920-s001.zip › jof-1814006-supplementary.pdf]

Complementary effects of dark septate endophytes and *Trichoderma* strains on growth and active ingredient accumulation of *Astragalus membranaceus* under drought stress

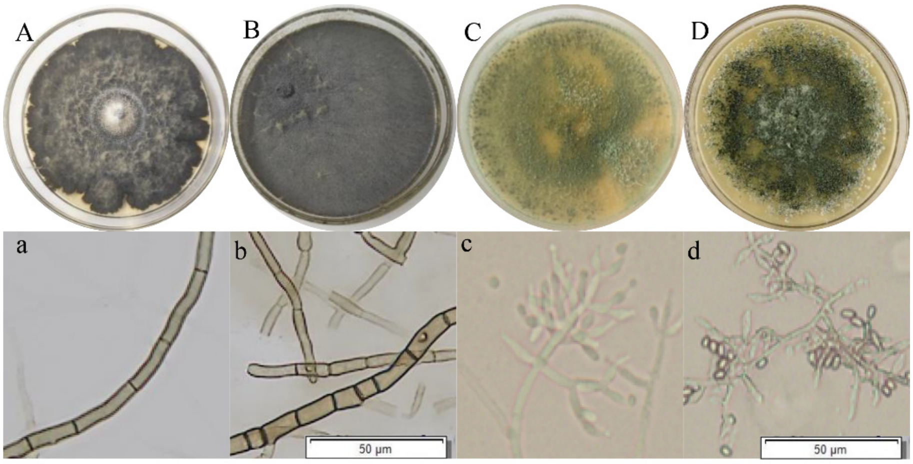

Figure S1: Colonies and microscopic morphology of DSE and *Trichoderma*. A(a)-D(d) indicate *Macrophomina pseudophaseolina* (A, a); *Paraphoma radicina* (B, b); *Trichoderma afroharzianum* (C, c); *Trichoderma longibrachiatum* (D, d). Scale bars = 50 µm.

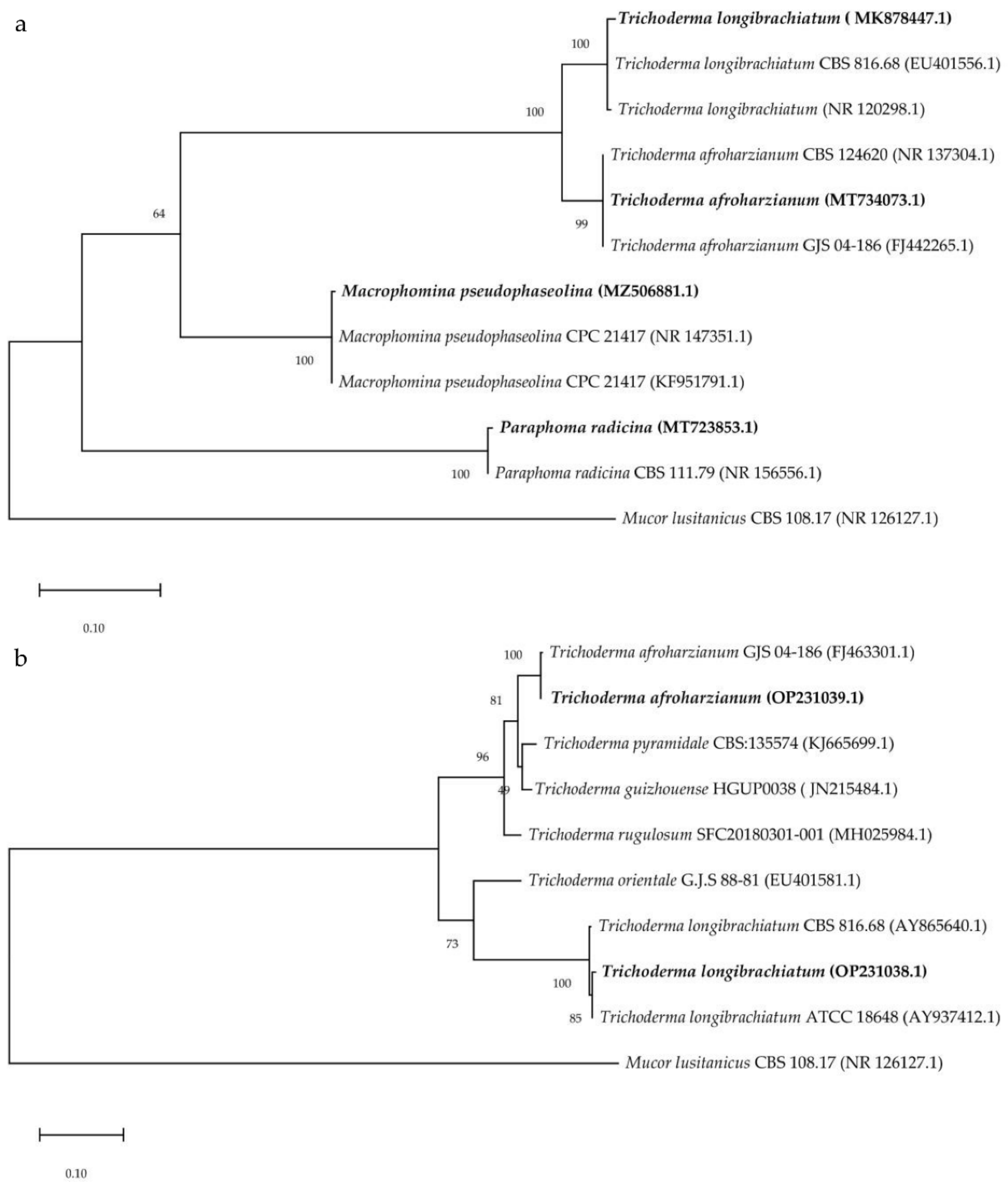

Figure S2: Maximum likelihood tree based on rDNA ITS region sequences of DSE and *Trichoderma* (**a**) and tef1 sequences of *Trichoderma* (**b**).

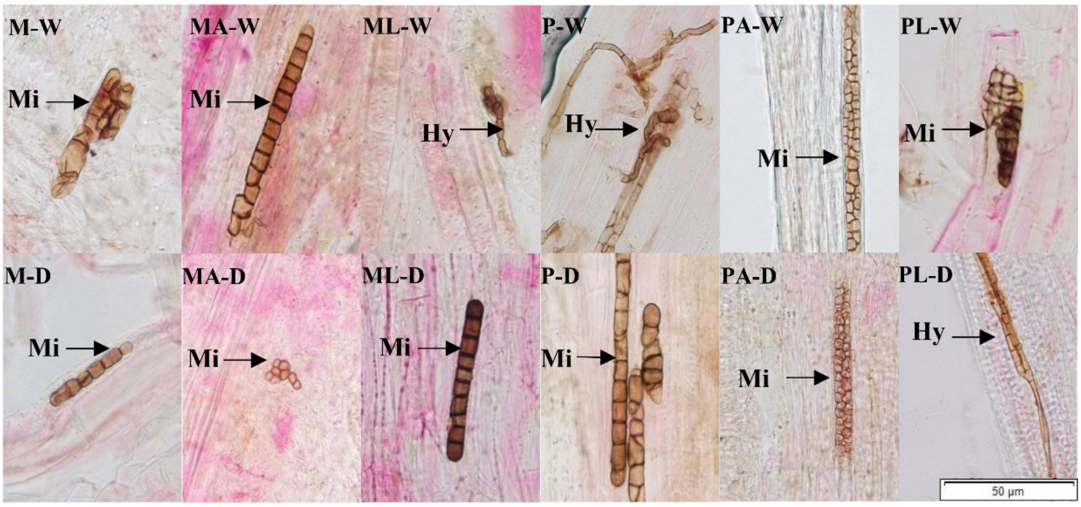

Figure S3: Colonization of dark septate endophytes (DSE) in the roots of *Astragalus mongholicus* four months after inoculation. W: Well-watered; D: drought stress; M: *Macrophomina pseudophaseolina*; P: *Paraphoma radicina*; MA: *M pseudophaseolina* + *Trichoderma afroharzianum*; ML: *M pseudophaseolina* + *Trichoderma longibrachiatum*; PA: *P radicina* + *T afroharzianum*; PL: *P radicina* + *T longibrachiatum*. Hy indicates DSE hyphae, Mi indicates DSE microsclerotia. Scale bars = 50 μm.

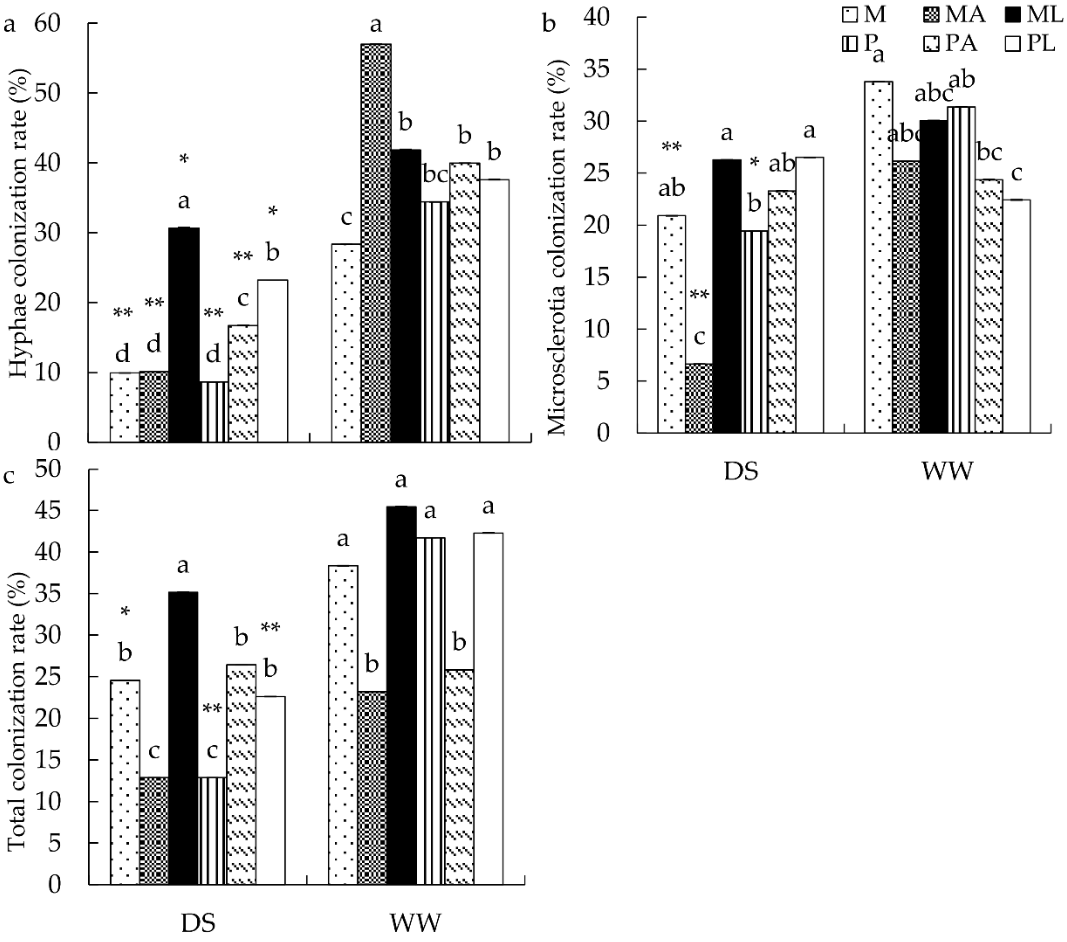

Figure S4: Hyphae colonization rate (**a**), microsclerotia colonization rate (**b**), and total colonization rate (**c**) of dark septate endophytes (DSE) in the roots of *Astragalus mongholicus* four months after inoculation. DS: drought stress; WW: Well-watered; M: *Macrophomina pseudophaseolina*; P: *Paraphoma radicina*; MA: *M pseudophaseolina* + *Trichoderma afroharzianum*; ML: *M pseudophaseolina* + *Trichoderma longibrachiatum*; PA: *P radicina* + *T afroharzianum*; PL: *P radicina* + *T longibrachiatum*.

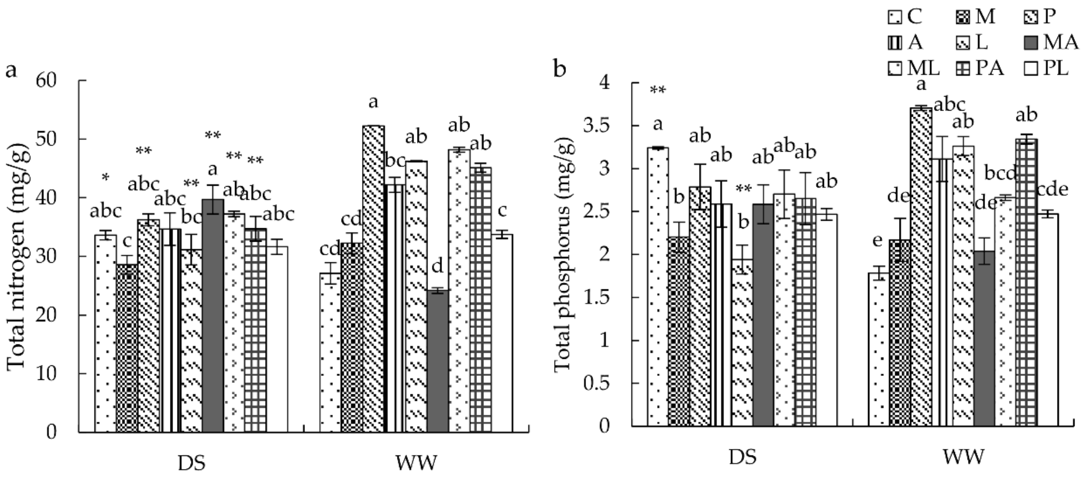

Figure S5: The effects of DSE inoculation, *Trichoderma* inoculation, and water conditions on total nitrogen (**a**) and total phosphorus (**b**) of *Astragalus mongholicus*. The error bars represent

the standard error of the mean. Different letters above the error bars indicate a significant difference at $p < 0.05$ by Tukey's test. DS, drought stress conditions; WW, well-watered conditions. C indicates non-inoculated control. M, P, A, L, MA, ML, PA, and PL indicate plants inoculated with *Macrophomina pseudophaseolina*; *Paraphoma radicina*; *Trichoderma afroharzianum; Trichoderma longibrachiatum*; *M. seudophaseolina + T. froharzianum; M. seudophaseolina + T longibrachiatum; P radicina + T afroharzianum; P radicina + T longibrachiatum.;*

Table S1. Analysis of variance (ANOVA) for the effects of water condition, DSE inoculation and *Trichoderma* inoculation on the growth of *Astragalus membranaceus*.

	Water		DSE		*Trichoderma*		Water x DSE		Water x *Trichoderma*		DSE x *Trichoderma*		Water x DSE x *Trichoderma*	
	F	*P*	*F*	*P*	*F*	*P*	*F*	*P*	*F*	*P*	*F*	*P*	*F*	*P*
Shoot biomass	68.65	**	0.20	NS	3.32	*	1.16	NS	5.02	*	2.64	*	0.97	NS
Root biomass	1792.56	**	52.62	**	24.22	**	7.63	**	38.65	**	48.81	**	15.78	**
Total root length	627.76	**	6.69	**	0.02	NS	3.37	*	0.18	NS	6.69	**	5.18	**
Root diameter	6.86	*	8.53	**	0.11	NS	0.19	NS	1.48	NS	0.93	NS	2.64	*
Leaf proline	3.20	NS	1.09	NS	4.31	*	4.47	*	7.49	**	6.55	**	7.48	**
Leaf soluble protein	20.35	**	10.93	**	23.10	**	0.14	NS	0.11	NS	6.43	**	0.76	NS
Leaf GSH	5172.99	**	597.279	**	467.52	**	1427.92	**	531.37	**	940.03	**	571.35	**
Leaf SOD	4.57	*	400.74	**	52.02	**	224.26	**	98.64	**	124.67	**	207.52	**
Root NR	0.35	NS	342.49	**	92.79	**	98.28	**	24.74	**	103.15	**	79.55	**
Root ASA	2.84	NS	352.61	**	132.58	**	521.72	**	98.34	**	92.80	**	117.22	**
Root GSH	35882.48	**	388.81	**	20276.50	**	2529.73	**	19860.31	**	21670.92	**	3321.76	**
Root IAA	18582.02	**	366.66	**	2859.47	**	879.54	**	2592.53	**	1190.29	**	4368.60	**
Calycosin-7-O -β-D-glucoside	40.95	**	108.00	**	11.91	**	15.86	**	36.69	**	53.00	**	15.23	**
Formononetin	3853.30	**	2608.35	**	98.37	**	870.26	**	265.27	**	499.71	**	251.55	**
Plant total N	44.73	**	11.57	**	7.21	**	17.03	**	14.11	**	39.68	**	32.55	**
Plant total P	1.63	NS	6.00	**	0.42	NS	3.19	NS	2.29	NS	3.69	*	7.89	**
U	12.95	**	105.95	**	71.92	**	50.32	**	10.05	**	24.09	**	57.05	**
ALP	51.22	**	1.23	NS	4.04	*	12.70	**	2.97	NS	9.89	**	1.11	NS
SOC	0.02	NS	3.70	*	4.27	*	0.19	NS	0.69	NS	2.62	NS	0.20	NS
SAP	0.41	NS	31.13	**	32.26	**	11.67	**	9.22	**	0.64	NS	2.51	NS
NH_4^+-N	3.04	NS	3.64	*	92.49	**	68.55	**	9.00	**	18.58	**	56.8	**
NO_3^--N	38.81	**	183.98	**	35.33	**	43.33	**	25.04	**	19.14	**	4.80	**

GSH: glutathione; SOD: superoxide dismutase; NR: nitrate reductase; ASA: ascorbic acid; MDA: malondialdehyde; IAA: indole-3-acetic acid; SOC = soil organic carbon; SAP = soil available phosphorus; ALP = soil alkaline phosphatase; U = urease; NH_4^+-N = soil ammonia nitrogen; NO_3^--N = soil nitrate nitrogen. * and ** indicate significance at $p \leq 0.05$ and $p \leq 0.01$, respectively.

Table S2. Correlation analysis of the growth indicators and soil properties of *Astragalus membranaceus* inoculated with DSE under drought stress condition.

DSE	SB	RB	RL	C	F	IAA	NR	SAP	ALP	U	NH_4^+-N	NO_3^--N
SB	1											
RB	0.292	1										
RL	0.273	**0.889****	1									
C	0.248	**0.814****	**0.787****	1								
F	0.297	0.526	0.420	-0.038	1							
IAA	-0.212	-0.331	-0.195	0.266	**-0.967****	1						
NR	0.449	**0.951****	**0.831****	**0.871****	0.410	-0.201	1					
SAP	0.008	0.366	0.391	**0.808****	-0.564	**0.715***	0.471	1				
ALP	0.341	**0.915****	**0.734***	**0.664***	**0.598***	-0.444	**0.842****	0.238	1			
U	0.358	**0.924****	**0.786****	0.542	**0.808****	**-0.662***	**0.843****	0.007	**0.913****	1		
NH_4^+-N	-0.083	0.117	0.210	**0.656***	**-0.769****	**0.891****	0.240	**0.945****	-0.040	-0.263	1	
NO_3^--N	-0.023	**-0.643***	**-0.639***	**-0.937****	0.276	-0.470	**-0.694***	**-0.940****	-0.509	-0.321	**-0.811****	1

SB: shoot biomass; RB: root biomass; RL: root length; C: calycosin-7-O-β-D-glucoside; F: formononetin; IAA: indole-3-acetic acid; NR: root nitrate reductase; SAP = soil available phosphorus; ALP = soil alkaline phosphatase; U = urease; NH_4^+-N = soil ammonia nitrogen; NO_3^--N = soil nitrate nitrogen. * and ** indicate significance at $p \leq 0.05$ and $p \leq 0.01$, respectively.

Table S3. Correlation analysis of the growth indicators and soil properties of *Astragalus membranaceus* inoculated with *Trichoderma* under drought stress condition.

Trichoderma	SB	RB	RL	C	F	IAA	NR	SAP	ALP	U	NH_4^+-N	NO_3^--N
SB	1											
RB	0.555	1										
RL	**0.583***	**0.703***	1									
C	0.049	**0.767****	0.469	1								
F	-0.067	**-0.752****	-0.423	**-0.994****	1							
IAA	**0.869****	**0.689***	**0.604***	0.091	-0.080	1						
NR	**0.787****	0.552	0.348	-0.052	0.061	**0.900****	1					
SAP	**-0.799****	-0.084	-0.304	0.422	-0.404	**-0.683***	**-0.658***	1				
ALP	0.362	0.498	0.307	0.465	-0.498	0.269	0.006	0.103	1			
U	**0.717***	**0.950****	**0.761****	**0.703***	**-0.702***	**0.706***	0.544	-0.228	**0.611***	1		
NH_4^+-N	**0.592***	**0.887****	**0.603***	**0.600***	**-0.610***	**0.633***	0.558	-0.114	0.538	**0.895****	1	
NO_3^--N	**0.729***	**0.842****	0.549	0.528	-0.543	**0.716***	**0.708***	-0.433	0.259	**0.860****	**0.830****	1

SB: shoot biomass; RB: root biomass; RL: root length; C: calycosin-7-O-β-D-glucoside; F: formononetin; IAA: indole-3-acetic acid; NR: root nitrate reductase; SAP = soil available phosphorus; ALP = soil alkaline phosphatase; U = urease; NH_4^+-N = soil ammonia nitrogen; NO_3^--N = soil nitrate nitrogen. * and ** indicate significance at $p \leq 0.05$ and $p \leq 0.01$, respectively.

Table S4. Correlation analysis of the growth indicators and soil properties of *Astragalus membranaceus* co-inoculated with DSE and *Trichoderma* under drought stress condition.

Co-inoculation	SB	RB	RL	C	F	IAA	NR	SAP	ALP	U	NH_4^+-N	NO_3^--N
SB	1											
RB	-0.016	1										
RL	-0.240	0.284	1									
C	-0.129	**0.889****	0.274	1								
F	**-0.519***	0.289	-0.050	**0.504***	1							
IAA	-0.151	-0.114	-0.103	0.232	0.164	1						
NR	-0.273	**0.452***	-0.252	**0.684****	**0.663****	**0.608****	1					
SAP	-0.284	**0.612****	**0.567***	**0.646****	0.416	-0.041	0.144	1				
ALP	-0.074	0.260	-0.103	0.379	**0.623****	0.020	0.349	0.378	1			
U	**-0.546***	0.201	0.036	**0.504***	**0.913****	**0.454***	**0.677****	**0.482***	**0.586***	1		
NH_4^+-N	0.019	**0.656****	-0.251	**0.604****	0.343	0.036	**0.658****	0.057	0.359	0.228	1	
NO_3^--N	0.438	0.026	**-0.486***	-0.288	-0.313	**-0.535***	-0.140	**-0.582***	-0.199	**-0.572***	0.417	1

SB: shoot biomass; RB: root biomass; RL: root length; C: calycosin-7-O-β-D-glucoside; F: formononetin; IAA: indole-3-acetic acid; NR: root nitrate reductase; SAP = soil available phosphorus; ALP = soil alkaline phosphatase; U = urease; NH_4^+-N = soil ammonia nitrogen; NO_3^--N = soil nitrate nitrogen. * and ** indicate significance at $p \leq 0.05$ and $p \leq 0.01$, respectively.
